# Function of the transforming growth factor-β1/c-Jun N-terminal kinase signaling pathway in the action of thalidomide on a rat model of pulmonary fibrosis

**DOI:** 10.3892/etm.2013.1457

**Published:** 2013-12-19

**Authors:** XUEJUN LIU, LI QIAN, HAOYU NAN, MIAO CUI, XIAOYAN HAO, YUFENG DU

**Affiliations:** Department of Geriatric Diseases, The First Hospital of Shanxi Medical University, Taiyuan, Shanxi 030001, P.R. China

**Keywords:** thalidomide, pulmonary fibrosis, c-Jun N-terminal kinase, α-smooth muscle actin

## Abstract

The aims of this study were to observe the effects of thalidomide on a rat model of pulmonary fibrosis, to determine the protein expression levels of phosphorylated c-Jun N-terminal kinase (p-JNK) and α-smooth muscle actin (α-SMA) and to explore the mechanism underlying the preventive effect of thalidomide on pulmonary fibrosis. Ninety healthy male Wistar rats (200±20 g) were randomly divided into control (N), model (M), SP600125 (SP), thalidomide (T) and SP600125 plus thalidomide (SP + T) groups. Pulmonary fibrosis models were established in groups M, SP, T and SP + T by the intratracheal instillation of bleomycin (BLM). A gavage of thalidomide was administered to the rats in groups T and SP + T once daily, whereas normal saline was administered to the rats in the other groups. The rats in the SP and SP + T groups were injected intraperitoneally with SP600125 following BLM administration, whereas the rats in the other groups received dimethyl sulfoxide. Rats were randomly sacrificed on days 7, 14 and 28. Pathological changes were examined by light microscopy using hematoxylin and eosin staining. Hydroxyproline (HYP) levels in the lung tissues were detected using alkaline hydrolysis. The protein expression levels of p-JNK and α-SMA were measured by immunohistochemical staining and western blot analysis. In group M, alveolitis was most serious on day 7 and then eased on day 14; marked pulmonary fibrosis was observed on day 28. The fibrosis was markedly attenuated in the SP + T group compared with that in group M. The HYP content increased gradually with time after BLM administration and peaked on day 28. On days 14 and 28, the HYP content was lower in groups T and SP than in group M (P<0.05). The expression levels of p-JNK protein and α-SMA were significantly lower in groups SP, T and SP + T than those in group M on day 14 (P<0.05). The expression level of α-SMA was lower in group SP + T than those in groups SP and T on days 14 and 28 (P<0.05). The expression level of p-JNK protein in group T was higher than those in groups SP and SP + T on days 14 and 28 (P<0.05). Thus, thalidomide eased the degree of BLM-induced pulmonary fibrosis in rats by downregulating p-JNK and α-SMA expression.

## Introduction

Pulmonary fibrosis is a chronic, progressive and lethal diffuse interstitial lung disease. It has re-emerged as a focus of scientific study, due to its increasing incidence (1). Transforming growth factor β1 (TGF-β1) is a profibrotic cytokine that has an important function in pulmonary fibrosis. The c-Jun N-terminal kinase (JNK) signaling pathway is a significant downstream kinase pathway in the TGF-β signaling pathway (2,3,4). The excessive activation of JNK is related to pulmonary fibrosis. Thalidomide is an effective bidirectional immunomodulatory agent that is able to suppress the generation of tumor necrosis factor-α (TNF-α) and inhibit collagen synthesis, in addition to having inhibitory effects on liver fibrosis and cirrhosis (5). However, whether thalidomide inhibits pulmonary fibrosis through the TGF-β1/JNK signaling pathway has yet to be elucidated. Furthermore, the mechanisms underlying the effects of thalidomide in pulmonary fibrosis remain unclear. In the present study, a bleomycin (BLM)-induced model of pulmonary fibrosis was used to determine whether thalidomide acted to reduce pulmonary fibrosis through the TGF-β1/JNK signaling pathway. The effects of thalidomide on the model of pulmonary fibrosis were observed using immunohistochemistry and western blotting. Simultaneously, the mechanisms underlying the effects of thalidomide were explored, in order to provide the basis for new clinical treatments for pulmonary fibrosis.

## Materials and methods

### Animals

Ninety male Wistar rats were used in this study. The study was carried out in strict accordance with the recommendations in the Guide for the Care and Use of Laboratory Animals (Institute of Laboratory Animal Resources Commission on Life Sciences, National Academy Press, Washington DC, 1996). The animal-use protocol was reviewed and approved by the Institutional Animal Care and Use Committee (IACUC) of the First Hospital of Shanxi Medical University (Taiyuan, China).

### Model of pulmonary fibrosis

Male Wistar rats were injected intraperitoneally with 3% chloral hydrate (1 ml/100 g). Following anesthetization, BLM (0.3 ml, 5 mg/kg) was intratracheally administered to the rats. The rats were placed in a vertical position and rotated for several times in order to distribute the drug in the lung tissues uniformly, and 0.3 ml dimethyl sulfoxide (Wuhan Boster Biological Technology, Ltd., Wuhan, China) solution was injected intraperitoneally on the same day.

### Treatment groups

Ninety healthy male Wistar rats (200±20 g) were randomly assigned into control (N), model (M), SP600125 (SP; a JNK inhibitor), thalidomide (T) and SP600125 plus thalidomide (SP + T) groups (n=18 per group). Pulmonary fibrosis models were established in groups M, SP, T and SP + T by the intratracheal injection of 5 mg/kg bleomycin (BLM) on the first day, as described above, whereas group N was injected with normal saline. The rats of groups T and SP + T were treated with a gavage of thalidomide (100 mg/kg) in saline (3 ml, 0.9%) once daily, whereas the rats in the other groups were administered a gavage of the same volume of saline without thalidomide. The rats of groups SP and SP + T were injected intraperitoneally with SP600125 (15 mg/kg, dissolved in DMSO) following BLM administration, whereas the rats in the other groups received DMSO alone. Rats were randomly sacrificed by abdominal aortic phlebotomy on days 7, 14 and 28 and their lung tissues were collected for analysis. Pathological changes were examined under a light microscope by hematoxylin and eosin (H&E) staining; hydroxyproline (HYP) was detected in the lung tissues by alkaline hydrolysis; and the expression levels of phosphorylated JNK (p-JNK) protein and α-SMA were measured by immunohistochemical staining and western blot analysis, as described below.

### H&E staining

Lung tissues were fixed in 10% (w/v) neutral-buffered formalin for 24 h, dehydrated in a graded ethanol series, and embedded in paraffin. Sequential 6-μm sections of the lungs were placed on slides and stained with H&E for morphological analysis using a standard protocol. The slides were then investigated under a light microscope.

### HYP assay

The collagen content in the lung homogenates was examined by HYP assay using a HYP detection kit from Nanjing Jiancheng Bioengineering Institute (Nanjing, China). All steps of the HYP assay were performed according to the manufacturer’s instructions. The absorbance of each sample at 550 nm was read with a microplate reader (Thermo Fisher Scientific, Waltham, MA, USA).

### Immunohistochemical assay

The lung tissues from the rats were fixed in 4% paraformaldehyde, dehydrated, embedded in paraffin and sectioned. The sections were incubated overnight at 4°C with a 1:200 dilution of mouse anti-mouse α-smooth muscle actin (α-SMA) monoclonal antibody (Wuhan Boster Biological Technology, Ltd.) or a 1:1,000 dilution of rabbit anti-mouse p-JNK monoclonal antibody (Cell Signaling Technology, Inc., Danvers, MA, USA). This procedure was followed by incubation with goat anti-mouse/goat anti-rabbit secondary antibody (Wuhan Boster Biological Technology, Ltd.) for 20 min at 37°C, and with avidin-biotin-conjugated horseradish peroxidase following the manufacturer’s instructions (Wuhan Boster Biological Technology, Ltd.). Samples were stained with hematoxylin for 30 sec subsequent to being fixed by dehydration. The immunohistochemical staining results were studied by computer image analysis (Image-Pro Plus 6.0; Photometrics, Tucson, AZ, USA). The positively stained gray value was determined and this was used as the mean value for statistical analysis. A low average gray value and a deep color indicated a high protein content.

### Western blot analysis

Lung tissues were homogenized in ice-cold radioimmunoprecipitation lysis buffer (Wuhan Boster Biological Technology, Ltd.). After centrifugation at 12,000 × g for 10 min at 4°C, the supernatant was collected, and the protein concentration was determined using a bicinchoninic acid protein assay kit (Boster Company, Wuhan, China). Proteins (30 μg) were separated by sodium dodecyl sulfate polyacrylamide gel electrophoresis, and transferred to polyvinylidene fluoride membranes. The blotted membranes were blocked with 5% bovine serum albumin in Tris-buffered saline with 0.1% Tween-20 (TBS-T), and incubated at 4°C overnight with a 1:200 dilution of mouse anti-mouse α-SMA monoclonal antibody (Wuhan Boster Biological Technology, Ltd.) or 1:1,000 dilution of rabbit anti-mouse p-JNK monoclonal antibody (Cell Signaling Technology, Inc.), or anti-β-actin antibodies (Wuhan Boster Biological Technology, Ltd.). After rinsing five times with TBS-T at 5-min intervals, the membranes were incubated for 2 h at 4°C a 1:5,000 dilution of horseradish peroxidase-labeled goat anti-mouse/goat anti-rabbit secondary antibody (BOSTER Company, Wuhan, China). Immunodetection was performed with enhanced chemiluminescence detection reagents (Applygen Technologies Inc., Beijing, China) and a chemiluminescence gel imaging system (FluorChem HD2; ProteinSimple, Santa Clara, CA, USA), with β-actin as the internal control.

### Statistical analysis

Results are presented as the mean ± standard error the mean. The groups were compared using one-way analysis of variance (ANOVA). P<0.05 was considered to indicate a statistically significant difference.

## Results

### Pathological changes

**Figure 1:**
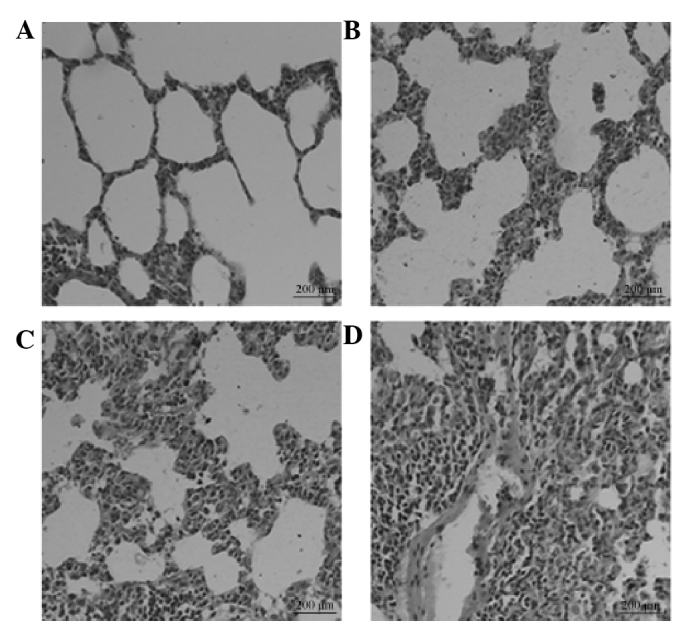
Hematoxylin and eosin staining of lung tissues in rats (magnification, ×100). (A) Group N (control); (B) Group M (model group) on day 7; (C) Group M on day 14; (D) Group M on day 28.

In group M, alveolitis was most severe on day 7 and then eased on day 14. However, marked pulmonary fibrosis was observed in this group on day 28 (Fig. 1). The degree of fibrosis in groups SP, T and SP + T was attenuated compared with that in group M, with the SP + T group exhibiting the most marked improvement.

### Hydroxyproline (HYP) levels

**Figure 2:**
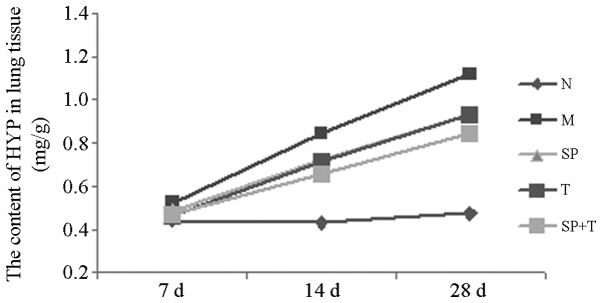
Hydroxyproline (HYP) levels in lung tissue. N, control; M, model; SP, SP600125; T, thalidomide; SP+T, SP600125 + thalidomide. The results for the SP group are not visible as those for group M overlap them.

No significant differences were observed among the HYP levels in group N on days 7, 14 and 28. The HYP level increased gradually with time and peaked on day 28 in groups M, SP, T and SP + T. On days 14 and 28, the HYP levels in groups M, SP, T and SP + T were significantly higher than that in group N (P<0.05); however, the levels in groups SP, T and SP + T were significantly lower than that in group M (P<0.05). On day 28, the HYP levels were significantly lower in the group SP + T than those in the other BLM-treated groups (P<0.05; Table I, Fig. 2).

### Immunohistochemical staining

**Figure 3:**
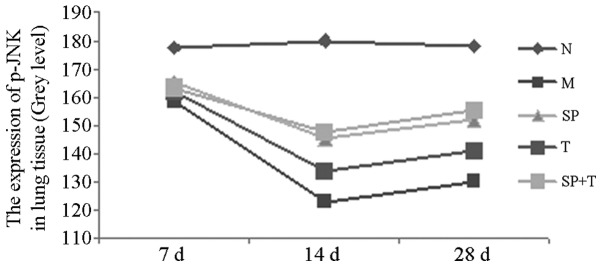
Protein expression of phosphorylated c-Jun N-terminal kinase (p-JNK) in lung tissue. N, control; M, model; SP, SP600125; T, thalidomide; SP+T, SP600125 + thalidomide.

**Figure 4:**
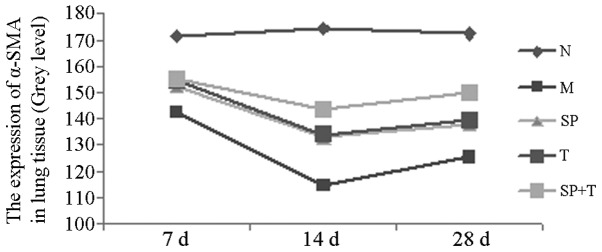
Protein expression of α-smooth muscle actin (α-SMA) in lung tissue. N, control; M, model; SP, SP600125; T, thalidomide; SP+T, SP600125 + thalidomide.

The protein expression levels of phosphorylated JNK (p-JNK) and α-smooth muscle actin (α-SMA) in group M were significantly higher than those in group N (indicated by a low average gray value), with the most marked difference on day 14 (P<0.05). The protein expression levels of p-JNK and α-SMA in groups SP, T and SP + T were significantly lower than those in group M, with the most notable differences on day 14 (P<0.05). The expression level of α-SMA in the SP + T group was lower than those in groups SP and T on days 14 and 28 (P<0.05). In addition, the expression of p-JNK protein in group T was significantly higher than those in groups SP and SP + T on days 14 and 28 (P<0.05; Tables II and III, Figs. 3 and 4).

### Western blot analysis

**Figure 5:**
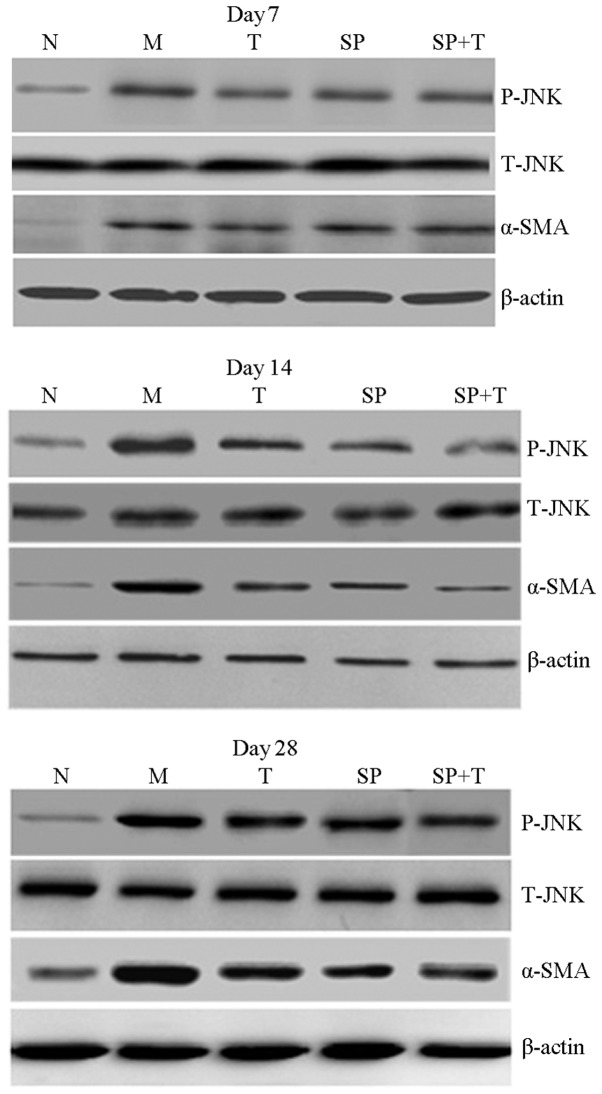
Assessment of phosphorylated c-Jun N-terminal kinase (p-JNK), T-JNK (total-JNK and α-smooth muscle actin (α-SMA) in the lung tissue of rats in different groups on days 7, 14 and 28. β-actin was used as the internal control. N, control; M, model; SP, SP600125; T, thalidomide; SP+T, SP600125 + thalidomide.

**Figure 6:**
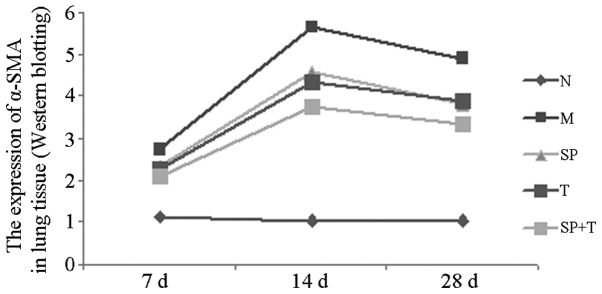
Protein expression of α-smooth muscle actin (α-SMA) in lung tissue (western blotting). N, control; M, model; SP, SP600125; T, thalidomide; SP+T, SP600125 + thalidomide.

**Figure 7:**
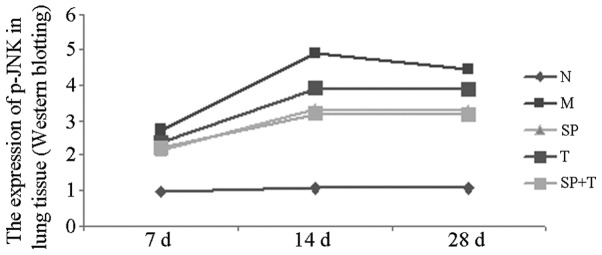
Protein expression of phosphorylated c-Jun N-terminal kinase (p-JNK) in lung tissue (western blotting). N, control; M, model; SP, SP600125; T, thalidomide; SP + T, SP600125 + thalidomide.

The protein expression levels of p-JNK and α-SMA in group M were significantly higher than those in group N, with the most marked difference on day 14 (P<0.05). In addition, the protein expression levels of p-JNK and α-SMA in groups SP, T and SP + T were significantly lower than those in group M and higher than those in group N (P<0.05). The expression of α-SMA in the SP + T group was lower than that in groups SP and T on days 14 and 28 (P<0.05), whereas the expression level of p-JNK protein in group T was significantly higher than that in groups SP and SP + T on days 14 and 28 (P<0.05). A significant positive correlation was observed between p-JNK protein and α-SMA levels in group M (r=0.858, P<0.05; Figs. 5–7).

## Discussion

Pulmonary fibrosis is a chronic, progressive and lethal diffuse interstitial lung disease that involves pulmonary interstitial substances, pulmonary alveoli and/or bronchioles. Pulmonary fibrosis is characterized by fibroblast proliferation and deposition of extracellular matrix materials. Effective therapeutic methods for the disease exist. The disease has high morbidity and mortality rates, with a five-year mortality rate of >50%. To improve the quality of life among patients with pulmonary fibrosis, the pathogenesis and treatment of the disease were investigated in the present study. Numerous studies have shown that thalidomide exhibits anti-inflammatory and immunomodulatory properties and exerts beneficial effects in myelofibrosis (6–8), hepatic fibrosis, renal fibrosis and pulmonary fibrosis. Furthermore, it has been demonstrated that thalidomide inhibits the production of inflammatory cytokines, including TNF-α, TGF-β1 and nuclear factor-κB (NF-κB), thereby reducing the degree of pulmonary fibrosis. However, the mechanisms underlying the effects of thalidomide in pulmonary fibrosis remain unclear. TGF-β1 is a profibrotic cytokine that has an important function in pulmonary fibrosis. The JNK signaling pathway is an important downstream signaling pathway of TGF-β1 (4). It has been indicated that the excessive activation of JNK is associated with pulmonary fibrosis (9,10). However, whether thalidomide acts through the TGF-β1/JNK signal transduction pathway remains unclear.

In recent years, the number of studies investigating signaling pathways has increased. It has been shown that TGF-β1 is an important fibrosis factor that is able to promote the transformation of epithelial cells to mesenchymal cells, induce lung fibroblasts to differentiate into muscle cells, upregulate the expression of α-SMA and collagen fibers and inhibit myofibroblast apoptosis (11). JNK is involved in an important signaling pathway downstream of TGF-β1 (10). Three kinds of JNK proteins exist, JNK1, JNK2 and JNK3; JNK1 and JNK2 are widely distributed in tissues, whereas JNK3 is distributed in the heart, brain and testes (13). In general, JNK predominantly exists in the cytoplasm. JNK is phosphorylated and activated when stimulated by upstream signals. The translocation of JNK into the nucleus and the activation of the nuclear transcription factor c-Jun enhance transcriptional activity. JNK is also able to activate the transcription factors activator protein-1 and Elk-1, further increasing the transcription of specific genes, proliferation, differentiation and apoptosis, which are involved in the regulation of many cellular activities. Hashimoto *et al* (9) demonstrated that the JNK signaling pathway was important in the differentiation of fibroblasts to myofibroblasts in the human lungs. Furthermore, the absence of the JNK1 gene may prevent pulmonary fibrosis in rats (10). SP600125 is a benzothiazole derivative and a specific inhibitor of JNK, with mechanisms involving reversing the ATP-competitive inhibitor and blocking JNK (14). In our previous experiments, administering SP600125 to Wistar rats with pulmonary fibrosis inhibited JNK (15). The reduced expression levels of α-SMA and collagen deposition in the lung tissues of rats with pulmonary fibrosis inhibited the differentiation of fibroblasts to myofibroblasts and decreased the degree of pulmonary fibrosis. Thus, the TGF-1/JNK signaling pathway has an important function in pulmonary fibrosis.

The results of the present study showed that only a low level of p-JNK protein expression was apparent in group N at each time point. However, in group M, p-JNK protein expression increased on day 7, reached its peak on day 14 and then decreased on day 28. The expression of a large quantity of α-SMA resulted in the substantial phosphorylation of JNK protein and activation of pulmonary fibrotic activity. Thus, the p-JNK protein may have an important function in the development of pulmonary fibrosis.

Thalidomide is a derivative of glutamic acid that has been used as a two-way immune regulator with anti-inflammatory, immunoregulatory and anti-angiogenic effects (16). Arai *et al* (17) demonstrated that thalidomide was able to reduce the levels of the vascular endothelial growth factor, TGF-β1 and α-SMA to prevent the occurrence of peritoneal fibrosis in rats. Thalidomide has also been shown to inhibit the TGF-β1-induced differentiation of lung fibroblasts into myofibroblasts and the synthesis of α-SMA and collagen. Furthermore, Chong *et al* (18) revealed that thalidomide downregulated the expression of inflammatory factors in animal experiments and reduced the degree of liver fibrosis, while Ye *et* al (19) demonstrated that thalidomide reduced levels of interleukin-8 and TNF. Thus, it has been indicated that thalidomide has therapeutic potential in the treatment of pulmonary fibrosis. Our previous study (20) showed that thalidomide inhibits the over-expression of type I collagen in pulmonary fibrosis rats via inhibition the JNK signaling pathway. Choe *et al* (5) demonstrated that thalidomide acted against liver fibrosis by inhibiting the TGF-β1/extracellular signal-regulated kinase 1/2 signal pathway. Our previous study (21) further indicated that thalidomide was able to downregulate the levels of TNF-α and TGF-β in the lungs of rats, and exhibit an attenuating effect on pulmonary fibrosis. In addition, Horton and Hallowell (22) proposed that thalidomide had potential as a drug for the treatment of idiopathic pulmonary fibrosis, due to its immune regulation and anti-angiogenic effects. A randomized trial conducted by the Johns Hopkins University showed that thalidomide was able to improve coughing in patients with idiopathic pulmonary fibrosis and enhance their quality of life (23). However, whether thalidomide has an important function in the occurrence and development of pulmonary fibrosis by the TGF-β1/JNK signaling pathway has yet to be elucidated. In the present study, using thalidomide and SP600125, a blocker of the JNK signaling pathway, to target pulmonary fibrosis in rats, the protein expression levels of p-JNK and α-SMA were observed in different groups to determine the mechanism by which thalidomide acted in pulmonary fibrosis.

The results of hematoxylin and eosin staining showed that the degree of fibrosis in the lung tissues of each rat was reduced in the model of pulmonary fibrosis following thalidomide injection. The HYP content in the lung tissues in group M was low on days 14 and 28, although higher than that in group N (P<0.05). Immunohistochemistry and western blot analysis showed that although the expression of α-SMA in group N lung tissues was high, it was lower than that in group M on days 14 and 28 (P<0.05). Thus, thalidomide reduced pulmonary fibrosis in rats.

The results of the immunohistochemistry and western blot analysis showed that p-JNK and α-SMA protein expression levels increased on day 7, peaked on day 14 and then declined on day 28 in the BLM-treated rats. The protein expression levels of p-JNK and α-SMA in group T were lower on days 14 and 28 than those in group M; however, they were higher than those of group N (P<0.05). Levels in groups SP and SP + T increased significantly compared with group T (P<0.05). Thalidomide reduced the activation of p-JNK and α-SMA protein expression; however, this effect was attenuated by the specific JNK inhibitor SP600125. Thus, thalidomide is indicated to act via the JNK signaling pathway. JNK has a key function in pulmonary fibrosis; however, it is not the only mechanism.

In conclusion, thalidomide is able to inhibit the JNK signaling pathway *in vivo*. This pathway has a key function in the treatment of pulmonary fibrosis. Given that the pathogenesis of pulmonary fibrosis is complicated, further studies are required to investigate the pathogenesis and treatment of pulmonary fibrosis.

## Figures and Tables

**Table I:** Hydroxyproline (HYP) levels.

		HYP (mg/g)		
		
Group	No.	7 days (n=6)	14 days (n=6)	28 days (n=6)
N	18	0.442±0.036	0.427±0.049	0.476±0.030
M	18	0.523±0.045^a^	0.847±0.086^a^	1.120±0.081^a^
SP	18	0.485±0.062	0.725±0.071^a^,^b^	0.931±0.070^a^,^b^
T	18	0.463±0.059	0.713±0.079^a^,^b^	0.934±0.080^a^,^b^
SP+T	18	0.467±0.050	0.659±0.074^a^,^b^	0.846±0.066^a^–^d^

Results are presented as the mean ± standard error of the mean. N, control; M, model; SP, SP600125; T, thalidomide; SP+T, SP600125 + thalidomide.

a P<0.05 vs. group N;

b P<0.05 vs. group M;

c P<0.05 vs. group SP;

d P<0.05 vs. group T.

**Table II:** Expression of phosphorylated c-Jun N-terminal kinase (p-JNK).

		p-JNK (gray level)		
		
Group	No.	7 days (n=6)	14 days (n=6)	28 days (n=6)
N	18	177.69±7.06	180.05±8.12	177.89±9.43
M	18	158.34±4.33^a^	123.03±8.29^a^	130.09±8.15^a^
SP	18	165.26±6.45^a^	145.44±9.44^a^,^b^	152.04±7.85^a^,^b^
T	18	161.91±7.18^a^	133.91±7.96^a^–^d^	141.09±10.26^a^–^d^
SP+T	18	163.52±6.73^a^	147.81±9.28^a^,^b^	155.40±7.62^a^,^b^

Results are presented as the mean ± standard error of the mean. N, control; M, model; SP, SP600125; T, thalidomide; SP+T, SP600125 + thalidomide.

a P<0.05 vs. group N;

b P<0.05 vs. group M;

c P<0.05 vs. group SP;

d P<0.05 vs. group T.

**Table III:** Expression of α-smooth muscle actin (α-SMA).

		α-SMA (gray level)		
		
Group	No.	7 days (n=6)	14 days (n=6)	28 days (n=6)
N	18	171.59±7.06	174.28±7.93	172.46±10.03
M	18	142.74±6.78^a^	114.27±8.06^a^	125.65±6.69^a^
SP	18	152.31±7.31^a^,^b^	132.81±7.29^a^,^b^	138.11±8.31^a^,^b^
T	18	154.42±9.35^a^,^b^	134.00±7.53^a^,^b^	139.30±7.46^a^,^b^
SP+T	18	155.16±7.29^a^,^b^	143.54±7.35^a^–^d^	149.94±9.56^a^–^d^

Results are presented as the mean ± standard error of the mean. N, control; M, model; SP, SP600125; T, thalidomide; SP+T, SP600125 + thalidomide.

a P<0.05 vs. group N;

b P<0.05 vs. group M;

c P<0.05 vs. group SP;

d P<0.05 vs. group T.
